# Beyond native reactivity: radical SAM enzymes as platforms for new-to-nature chemistry

**DOI:** 10.1039/d6cb00249h

**Published:** 2026-09-11

**Authors:** Xuxue Liu, Xiangyang Gao, Sasipa Booranamonthol, Peng Wu, Rung-Yi Lai, Qi Zhang

**Affiliations:** a National Engineering Research Center for Carbohydrate Synthesis, College of Chemistry and Materials, Jiangxi Normal University Nanchang 330022 China qizhang_chem@fudan.edu.cn; b School of Chemistry, Institute of Science, Suranaree University of Technology Nakhon Ratchasima 30000 Thailand rylai@sut.ac.th

## Abstract

Radical *S*-adenosylmethionine (rSAM) enzymes constitute one of the largest and most versatile enzyme superfamilies in biology, catalyzing diverse radical-mediated reactions essential for metabolism, cofactor biosynthesis, nucleic acid modification, and natural product formation. Central to their chemistry is the reductive cleavage of *S*-adenosylmethionine (SAM) by an iron–sulfur cluster, generating the highly reactive 5′-deoxyadenosyl radical (5′-dAdo˙), which initiates a broad range of challenging transformations. Recent advances have expanded the reactivity of rSAM enzymes beyond their natural roles, revealing unprecedented mechanistic flexibility and synthetic potential. Photochemical activation strategies now enable light-driven reduction of [4Fe–4S] clusters in the presence of biological or chemical photosensitizers, thereby initiating radical formation. In parallel, cobalamin-dependent radical SAM methyltransferases have opened new avenues for ethyl and fluoromethyl transfer chemistry using corresponding SAM analogues. Notably, the use of a strategically engineered, stable analogue, such as 7-deazaadenine-tetrazole-substituted F-SAM (F-7dz-tSAM), has successfully overcome inherent cofactor degradation. Noncanonical radical reactivity, exemplified by ArsL-catalyzed C–As bond formation and NosL-mediated photoinduced trifluoromethylation, demonstrates the capacity of rSAM enzymes to perform new-to-nature transformations. Lastly, the repurposing of rSAM enzymes for formylglycine generation has enabled orthogonal aldehyde-tag formation for bioorthogonal protein labeling, further expanding their utility in chemical biology. Collectively, these advances establish rSAM enzymes as versatile platforms for radical biocatalysis, chemical biology, and new-to-nature chemistry.

## Abstract 2

Radical SAM enzymes are emerging as versatile platforms for new-to-nature chemistry. Photochemical activation, SAM analogues, and enzyme engineering expand their reactivity and biocatalytic potential.

## Graphical abstract

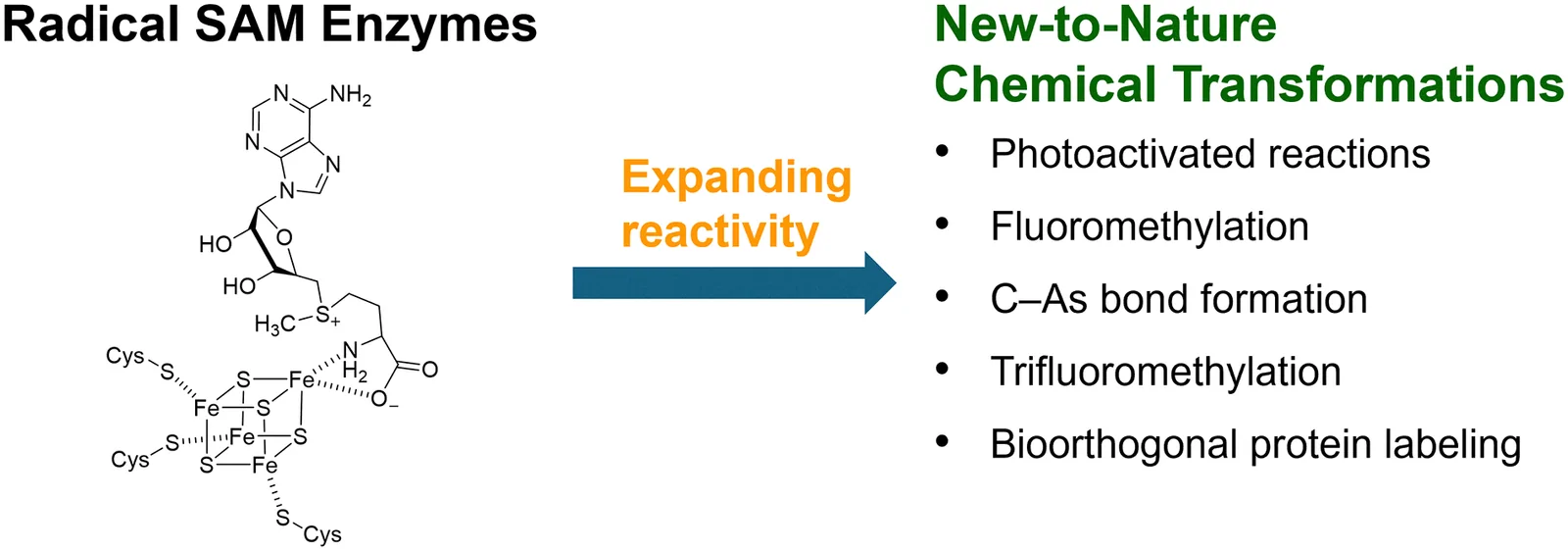

## 1. Introduction

Nature relies on radical chemistry to catalyze transformations that are thermodynamically or kinetically inaccessible through polar mechanisms. Enzymes that generate and control radical intermediates can activate otherwise inert chemical bonds, thereby enabling the construction of complex molecular architectures under mild physiological conditions. Among these enzymatic systems, the radical *S*-adenosylmethionine (rSAM) enzyme superfamily represents one of the most abundant, diverse, and chemically versatile catalytic families in biology.^1–3^ These enzymes mediate diverse reactions critical for metabolism,^4^ cofactor biosynthesis,^5^ nucleic acid modification,^6–9^ and natural product biosynthesis,^1,2^ highlighting their broad biological and chemical significance.

The rSAM superfamily was first recognized in 2001 through the pioneering work of Sofia and co-workers, who employed bioinformatic analysis to identify a conserved CxxxCxxC motif in a group of enzymes that share a common dependence on SAM.^10^ In this motif, three cysteine thiolates coordinate to three irons of a [4Fe–4S] cluster, while SAM binds to the fourth, unique iron in a bidentate fashion through its amino and carboxylate groups. Subsequent advances in bioinformatics, structural biology, and enzymology have revealed an exceptionally large and chemically diverse enzyme superfamily distributed across bacteria, archaea, and eukaryotes.^11^ This remarkable expansion underscores the evolutionary success of the rSAM fold and its ability to support an enormous range of radical-mediated transformations.

To date, rSAM enzymes have been associated with more than one hundred distinct transformations, including hydrogen abstraction, methylation, sulfur insertion, rearrangement, decarboxylation, desaturation, and carbon–carbon bond formation.^1–3^ Representative examples include BioB and LipA in sulfur insertion,^12–15^ HydE and HydG in [FeFe]-hydrogenase maturation,^16–18^ HemN and NirJ in tetrapyrrole cofactor biosynthesis,^19–21^ RlmN and Cfr in RNA modification,^22–25^ and complex rearrangement reactions, exemplified by MoaA and ThiC.^26–32^ Collectively, these reactions illustrate the capacity of the rSAM scaffold to generate and control highly reactive intermediates while maintaining remarkable chemo-, regio-, and stereoselectivity. rSAM enzymes also play prominent roles in natural product biosynthesis, where they catalyze chemically demanding transformations such as carbon–carbon and thioether bond formation, epimerization, sulfur insertion, and dehydrogenation.^33–40^ Their roles are particularly prominent in ribosomally synthesized and post-translationally modified peptides, in which radical-mediated tailoring generates structurally constrained and functionally diverse products. These natural transformations provide a rich mechanistic foundation for repurposing rSAM enzymes toward non-native substrates and new-to-nature chemistry.

The catalytic diversity of the rSAM superfamily has prompted growing efforts to redirect these enzymes beyond their native biological roles. Recent progress has occurred at several distinct levels. First, external electron-delivery strategies, particularly photochemical reduction, provide greater control over radical initiation and can improve catalytic turnover. Second, SAM analogue engineering expands the identity of transferable groups and enables reactions that are inaccessible with the native cofactor. Third, the catalytic promiscuity or engineering of selected rSAM enzymes has enabled noncanonical bond-forming reactions and site-selective biomolecular modification. Finally, advances in cofactor regeneration, reaction engineering, and protein design are beginning to address the operational limitations of rSAM catalysis. In this Review, we examine these developments within a unified framework encompassing electron-delivery control, SAM analogue engineering, enzyme repurposing, and emerging synthetic applications. A progression from native radical chemistry to programmable new-to-nature rSAM catalysis was summarized in Fig. 1.

**Fig. 1:**
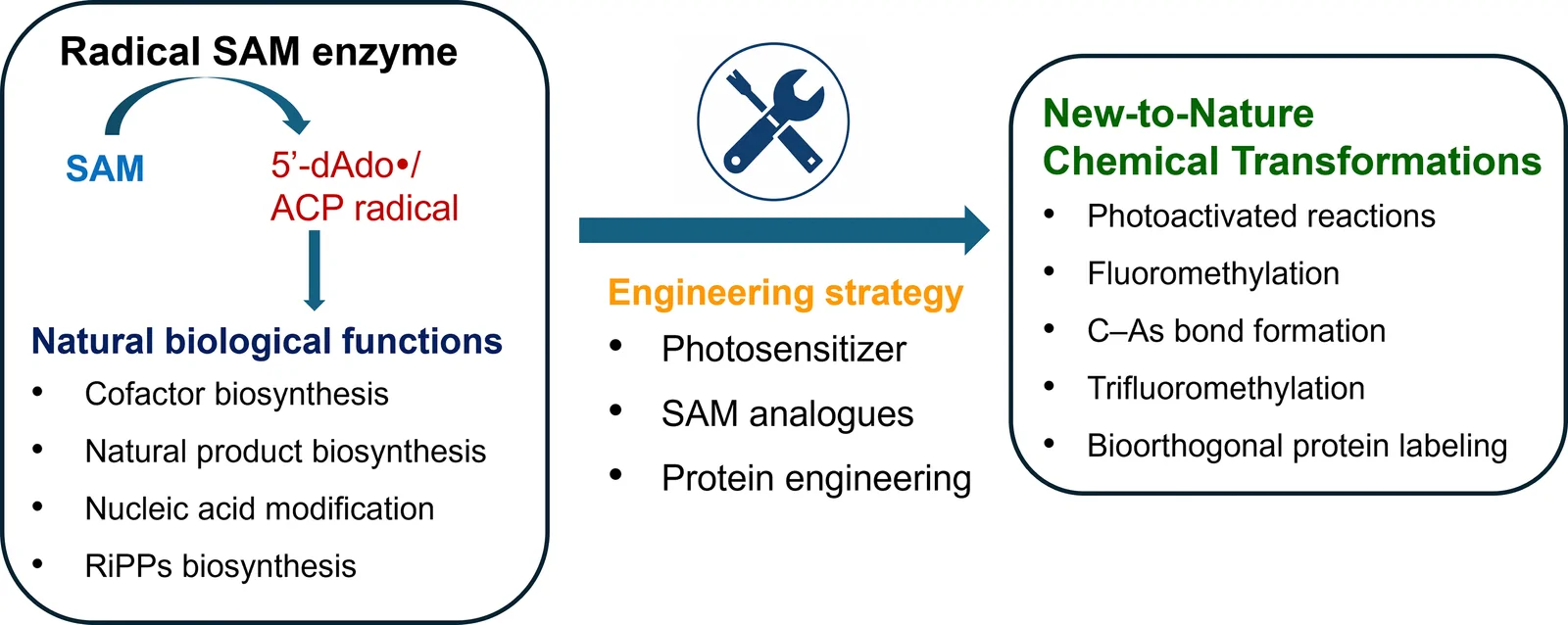
Overview of recent advances in radical *S*-adenosylmethionine enzymes. Radical *S*-adenosylmethionine (rSAM) enzymes generate either the 5′-deoxyadenosyl radical (5′-dAdo˙) or the 3-amino-3-carboxypropyl (ACP) radical from *S*-adenosylmethionine (SAM) to mediate diverse biological functions, including cofactor biosynthesis, natural product biosynthesis, nucleic acid modification, and RiPP biosynthesis. Recent engineering strategies, including the use of photosensitizers, SAM analogue engineering, and protein engineering, have expanded the catalytic repertoire of rSAM enzymes, enabling new-to-nature chemical transformations such as photoactivated reactions, fluoromethylation, C–As bond formation, trifluoromethylation, and bioorthogonal protein labeling.

## 2. Radical initiation and diversification in radical SAM enzymes

Despite their functional diversity, most characterized rSAM enzymes share a common radical-initiation framework in which SAM binds to the unique iron of a [4Fe–4S] cluster through its amino and carboxylate groups. Reduction of the cluster from the [4Fe–4S]^2+^ to the [4Fe–4S]^1+^ state promotes inner-sphere electron transfer to SAM and homolytic cleavage of the S–C5′ bond, producing methionine and the 5′-deoxyadenosyl radical (5′-dAdo˙). The regioselectivity of this cleavage arises from the interplay between the electronic structure of reduced SAM and the active-site environment. One-electron reduction generates a Jahn–Teller-active R_3_S^0^ state, in which distortion of the sulfonium center favors localization of the antibonding electron into a particular S–C bond. Importantly, the Jahn–Teller effect enables selective bond weakening but does not itself determine which S–C bond is cleaved; this selectivity is imposed by the enzyme active site through conformational constraints on bound SAM. During canonical catalysis, conformational changes associated with electron transfer favor localization of the distortion along the S–C5′ bond, thereby promoting formation of 5′-dAdo˙.^41^ The resulting radical typically initiates catalysis through hydrogen-atom abstraction from the substrate (Fig. 2).^41^

**Fig. 2:**
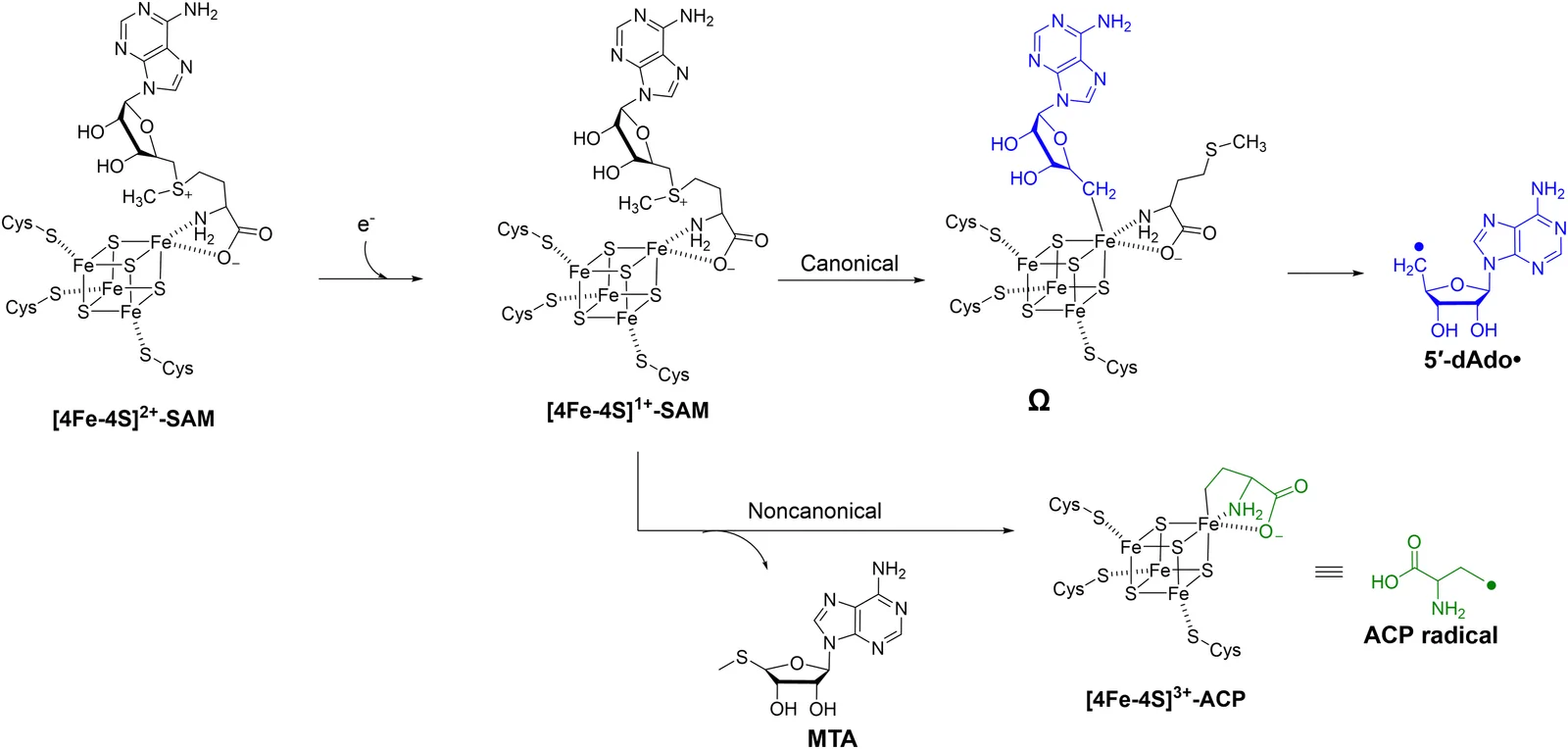
Canonical and noncanonical modes of SAM cleavage in radical SAM enzymes. In the canonical pathway, SAM coordinates to the unique iron of the [4Fe–4S]^2+^ cluster. One-electron reduction generates the catalytically active [4Fe–4S]^1+^–SAM complex and promotes cleavage of the S–C5′ bond to produce the 5′-deoxyadenosyl radical (5′-dAdo˙). In selected noncanonical rSAM systems, alternative binding geometries favor cleavage of the Cγ–S bond, generating the 3-amino-3-carboxypropyl (ACP) radical.

Spectroscopic and kinetic studies have revealed that SAM cleavage can proceed through a discrete organometallic intermediate known as Ω (Fig. 2). First identified in pyruvate formate-lyase activating enzyme, Ω contains a covalent Fe–C5′ bond between the unique iron of the [4Fe–4S] cluster and the adenosyl fragment.^42^ This intermediate transiently captures the nascent 5′-dAdo radical before homolysis of the Fe–C bond releases the reactive species for substrate activation. The ability of the cluster to transiently stabilize a carbon-centered radical provides an important mechanistic basis for controlling and potentially redirecting rSAM radical reactivity.

The 5′-dAdo radical is not the only radical species accessible through rSAM-dependent SAM cleavage. In the Dph1–Dph2 system, SAM is cleaved at the Cγ–S bond to generate 5′-methylthioadenosine (MTA) and the 3-amino-3-carboxypropyl (ACP) radical (Fig. 2), which is subsequently transferred to elongation factor 2 during diphthamide biosynthesis.^43,44^ Structural and spectroscopic studies support the formation of an organometallic ACP–iron intermediate and indicate that an alternative SAM-binding orientation positions the Cγ–S bond near the unique iron of the cluster.^45^ These findings further illustrate how the active-site-controlled orientation of SAM can redirect Jahn–Teller-enabled cleavage toward an alternative S–C bond, thereby altering the identity of the radical intermediate.

The potential to alter the identity of the radical generated from SAM has also been demonstrated in cryogenic mechanistic studies of HydG, an rSAM enzyme involved in [FeFe]-hydrogenase maturation.^46–49^ Under cryogenic photoirradiation, the HydG [4Fe–4S]^1+^–SAM complex undergoes S–C bond cleavage to generate a methyl radical (˙CH_3_).^50^ When SAM is replaced with the analogue *S*-adenosyl-l-ethionine (SAE), the corresponding ethyl radical (˙CH_2_CH_3_) is generated instead (Fig. 3).^51^ These studies demonstrate both that the HydG active site can accommodate a SAM analogue to diversify the radical species generated and that photoirradiation can directly trigger reductive S–C bond cleavage in the SAM–cluster complex. Although these reactions were observed under nonphysiological cryogenic conditions and do not themselves represent catalytic synthesis, they reveal the electronic flexibility of the SAM–cluster complex and highlight how SAM orientation and active-site electrostatics govern the regioselectivity of S–C bond cleavage. Together with the ACP-generating pathway, these findings establish SAM binding geometry and SAM analogue structure as key determinants of radical identity.^52^

**Fig. 3:**
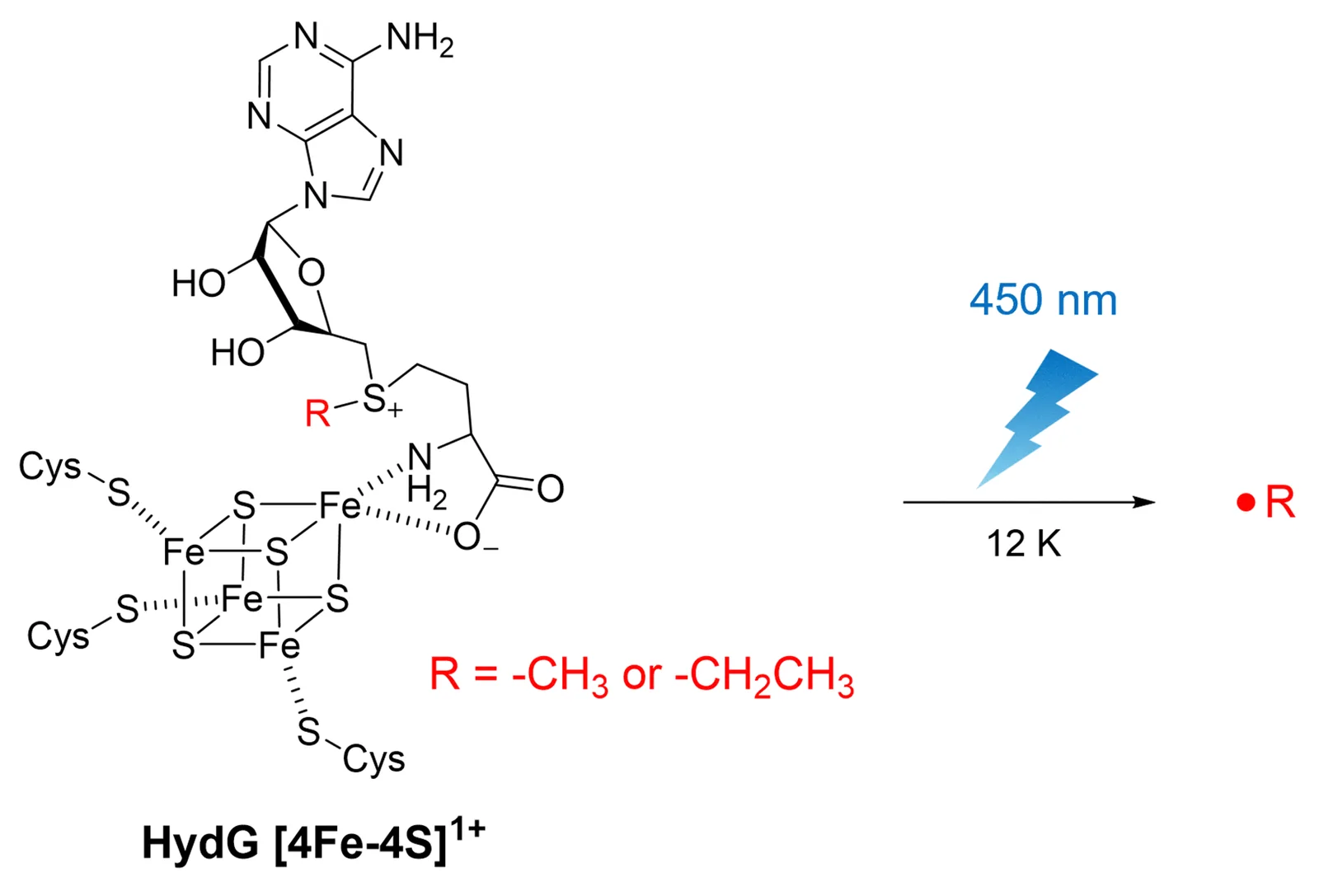
SAM analogue-dependent diversification of photoinduced radical generation in HydG. Under cryogenic photoirradiation at 12 K, the reduced HydG [4Fe–4S]^1+^ complex bound to SAM undergoes S–C bond cleavage to generate a methyl radical (˙CH_3_). Replacement of SAM with *S*-adenosyl-l-ethionine (SAE) leads to formation of the corresponding ethyl radical (˙CH_2_CH_3_), demonstrating that modification of the SAM substituent can alter the identity of the radical generated within the enzyme active site.

Collectively, these studies show that rSAM enzymes can access multiple carbon-centered radicals through changes in SAM cleavage regiochemistry, SAM analogue structure, and external activation conditions. Although several of these observations were initially made in mechanistic rather than preparative settings, they provide the conceptual basis for engineering radical identity and initiation in synthetic rSAM catalysis. The following section examines how light-driven electron delivery has translated this mechanistic potential into catalytic activation strategies.

## 3. Photochemical control of radical SAM catalysis

Traditional activation of rSAM enzymes requires reduction of the catalytic [4Fe–4S]^2+^ cluster to its [4Fe–4S]^1+^ state, which initiates electron transfer to SAM and generates the 5′-dAdo˙. The catalytic [4Fe–4S] clusters of rSAM enzymes typically exhibit reduction potentials ranging from −400 to −750 mV,^53^ depending on the protein environment and the presence of auxiliary clusters. In most *in vitro* studies, this reduction is achieved through either chemical or biological methods. Although the physiological electron-transfer partner of many rSAM enzymes remains unknown, the flavodoxin/flavodoxin reductase/NADPH (Fld/Fpr/NADPH) system has been successfully employed as a surrogate, operating at redox potentials around −400 to −450 mV.^53^ This system provides a physiologically relevant but relatively weak reducing environment, and its multicomponent nature, dependence on protein–protein interactions, and limited experimental controllability restrict its practical utility. Therefore, sodium dithionite is often used as a convenient chemical reductant because of its stronger reduction activity (*E*°′ ≈ −660 mV).^53^ However, sodium dithionite is chemically unstable in aqueous solution and can also intercept reactive radical intermediates, potentially leading to undesired side products.^54,55^ More recently, titanium(iii) citrate has emerged as another effective reductant for cobalamin-dependent rSAM enzymes; however, it is not universally effective.^56^ These energetic and practical limitations have motivated the development of alternative activation strategies capable of delivering more controlled and tunable reduction potentials. Among these, light-driven photoinitiation has emerged as a promising approach, employing photoinduced electron transfer to reduce rSAM iron–sulfur clusters and initiate catalysis. An early effort in this direction employed 5-deazariboflavin, a structural analog of flavin, as a photoreductant for rSAM enzymes in spectroscopic studies to identify the organometallic Ω intermediate.^42^ Although capable of promoting cluster reduction, its low quantum efficiency and limited reducing power curtailed broader application. Nevertheless, this pioneering work demonstrated the feasibility of photochemical activation and laid the foundation for the development of more efficient and tunable photoinitiation systems.

In 2023, Huang *et al.* introduced a genetically encoded photosensitizer protein (PSP2) as a biologically integrated photoreductant capable of directly reducing [4Fe–4S] clusters.^57^ PSP2, derived from a superfolder yellow fluorescent protein, contains a benzophenone–imidazolinone chromophore that forms a super-reducing radical (*E*° ≈ −1.14 V) under 405 nm irradiation in the presence of a sacrificial electron donor such as NADH.^58^ Using BtrN,^59^ an rSAM enzyme involved in the butirosin biosynthetic pathway, was shown to effectively reduce both the catalytic and auxiliary [4Fe–4S]^2+^ clusters, including the dithionite-inaccessible auxiliary cluster (*E*° ≈ −765 mV),^60^ to the [4Fe–4S]^1+^ state, as monitored by electron paramagnetic resonance (EPR) spectroscopy.^61^ Photoreduced BtrN catalyzed the conversion of 2-deoxy-*scyllo*-inosamine to its oxidized product, 3-amino-2,3-dideoxy-*scyllo*-inosose, with the formation of 5′-deoxyadenosine (5′-dA), confirming productive SAM cleavage (Fig. 4). Importantly, PSP2 also activated *Ph*Dph2,^62^ a noncanonical rSAM enzyme involved in diphthamide biosynthesis. In mechanistic studies performed in the absence of the substrate protein *Ph*EF2, EPR spectroscopy at 12 K revealed signals corresponding to both the [4Fe–4S]^1+^ cluster and the PSP2 radical, followed by SAM cleavage to yield 5′-methylthioadenosine (MTA). Upon addition of *Ph*EF2, ACP modification of His597 on *Ph*EF2 was observed, confirming enzymatic turnover (Fig. 4). Collectively, these results highlight the broad utility of the PSP2 radical as a biocompatible photoreductant for activating diverse rSAM enzymes.

**Fig. 4:**
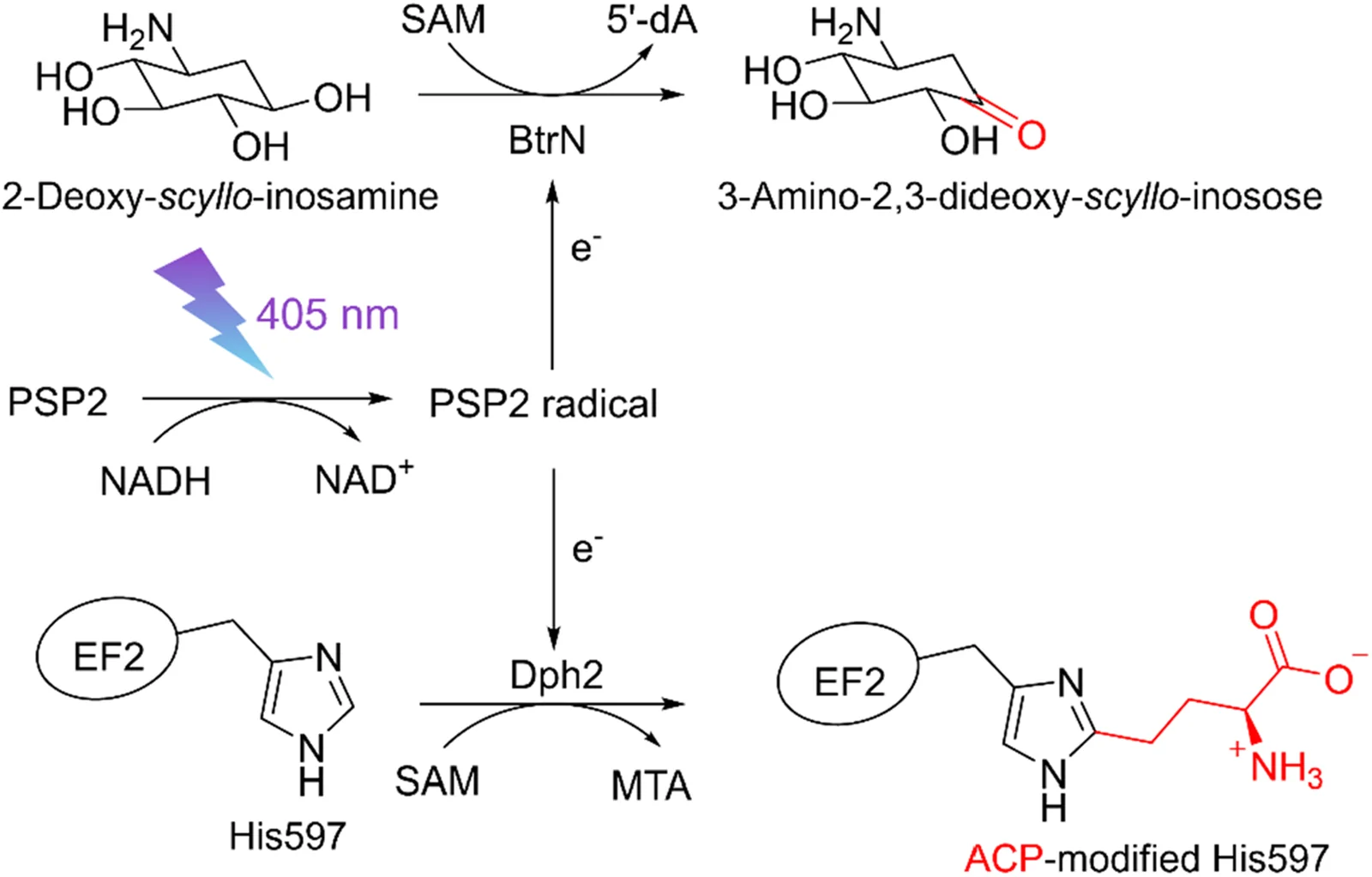
Photoreduced PSP2 activates rSAM enzymes. Upon 405 nm irradiation, PSP2 is converted into a super-reducing PSP2 radical using NADH as a sacrificial electron donor. The PSP2 radical transfers electrons to the [4Fe–4S] clusters of BtrN and Dph2, generating their catalytically active reduced states and initiating SAM cleavage. In BtrN, productive turnover is evidenced by the formation of 5′-deoxyadenosine (5′-dA), whereas in Dph2, cleavage of the Cγ–S bond of SAM produces 5′-methylthioadenosine (MTA). In the presence of their respective substrates, the corresponding enzymatic products are formed.

Building on this protein-based approach, Li *et al.* later (2025) developed a complementary light-driven catalytic platform that employs small-molecule photosensitizers to initiate rSAM reactions.^63^ Using *E. coli* ThiH as a model rSAM enzyme,^64,65^ several organic dyes were screened as photosensitizers under blue-light irradiation (450 nm) in the presence of NADH as a sacrificial electron donor.^66–70^ Among these, eosin Y (EY) proved to be the most effective (Fig. 5A), achieving complete conversion of l-tyrosine with a total turnover number (TTN) of 50, compared to a modest 10–15% yield obtained with sodium dithionite (DTH). Encouraged by this result, the EY system was subsequently applied to a variety of rSAM enzymes, including l-tryptophan lyase NosL (involved in nosiheptide biosynthesis),^71–73^ ArsS (involved in arsenosugar biosynthesis),^74^ diol dehydratase AprD4 (involved in apramycin biosynthesis),^75–78^ HemN (involved in heme biosynthesis),^79,80^ BlsE (involved in blasticidin E biosynthesis),^81–84^ and the noncanonical ArsL (involved in arsinothricin biosynthesis) (Fig. 5B).^85–87^ Compared with control reactions using DTH, most photoreduced reactions exhibited a 5- to 10-fold increase in yield and TTN. Collectively, these findings demonstrate that the EY-mediated photoreduction strategy is a broadly applicable and highly efficient activation method for rSAM enzymes, yielding catalytic performance superior to that achieved with the conventional DTH reductant.

**Fig. 5:**
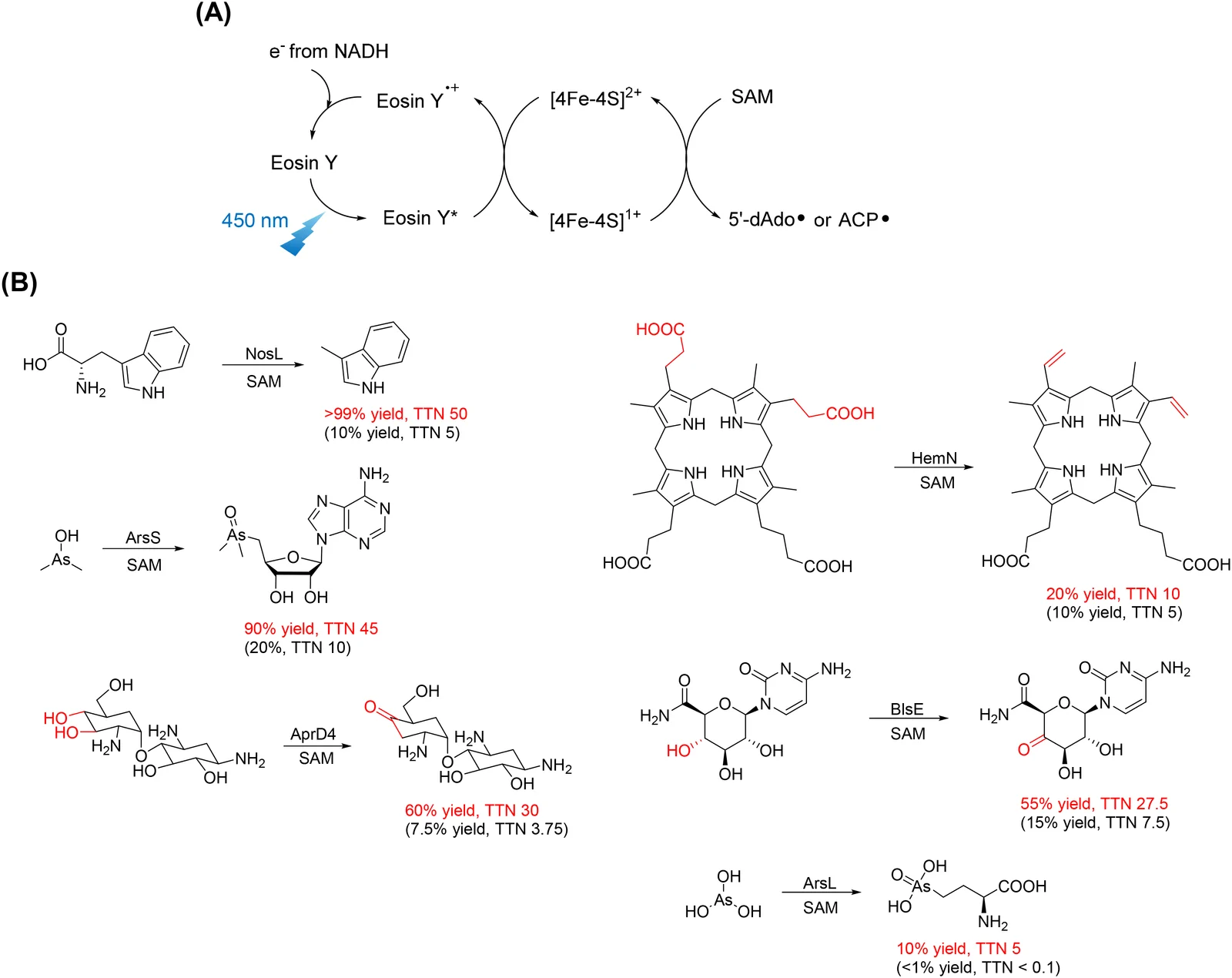
(A) General mechanism of SAM reductive cleavage under photocatalytic conditions. Upon irradiation, Eosin Y is excited to Eosin Y*, which transfers an electron to the [4Fe–4S]^2+^ cluster of an rSAM enzyme, promoting S–C bond cleavage to generate the corresponding 5′-deoxyadenosyl (5′-dAdo˙) or 3-amino-3-carboxypropyl radical (ACP˙). The resulting Eosin Y radical cation is subsequently reduced back to the ground-state dye by NADH, serving as a sacrificial reductant. (B) The generality of Eosin Y–mediated photocatalytic activation across diverse rSAM enzymes, including l-tryptophan lyase NosL involved in nosiheptide biosynthesis, ArsS in arsenosugar biosynthesis, diol dehydratase AprD4 in apramycin biosynthesis, HemN in heme biosynthesis, BlsE in blasticidin E biosynthesis, and the noncanonical ArsL in arsinothricin biosynthesis. Reaction yields and total turnover numbers (TTN) obtained under light-driven conditions exceed those achieved with the conventional chemical reductant dithionite (values in parentheses).

Interestingly, the EY-photoreduction system can also be employed to investigate the reaction mechanisms of rSAM enzymes, particularly with respect to redox stoichiometry, which is challenging to study using DTH. In redox-neutral reactions, no external reductant is required because the substrate-derived radical intermediate returns an electron to the oxidized [4Fe–4S]^2+^ cluster. This electron transfer regenerates the catalytically active [4Fe–4S]^1+^ state, while oxidation of the radical intermediate leads to product formation (Fig. 6). Using this system, the authors demonstrated that ArsS efficiently catalyzes the adenosylation of DMAs^III^ without NADH,^74^ generating 5′-deoxy-5′-dimethylarsinoyladenosine (DDMAA) (Fig. 6A). Furthermore, the EY system was shown to redirect the catalytic pathway of the NosL-catalyzed adenosylation reaction on an l-tryptophan analogue, 2-methyl-3-(indolyl)acrylic acid (MIAA).^88,89^ In the absence of NADH, the reaction predominantly produced the redox-neutral alkene product 1 (Fig. 6B), illustrating that modulation of reductant availability can influence product selectivity. Conversely, with the addition of NADH, the radical intermediate gains an electron, followed by protonation to form the alkane product 2. These findings demonstrate the potential of the photocatalytic EY system as a controllable platform for probing and modulating the redox dependence and catalytic outcomes of rSAM enzyme reactions.

**Fig. 6:**
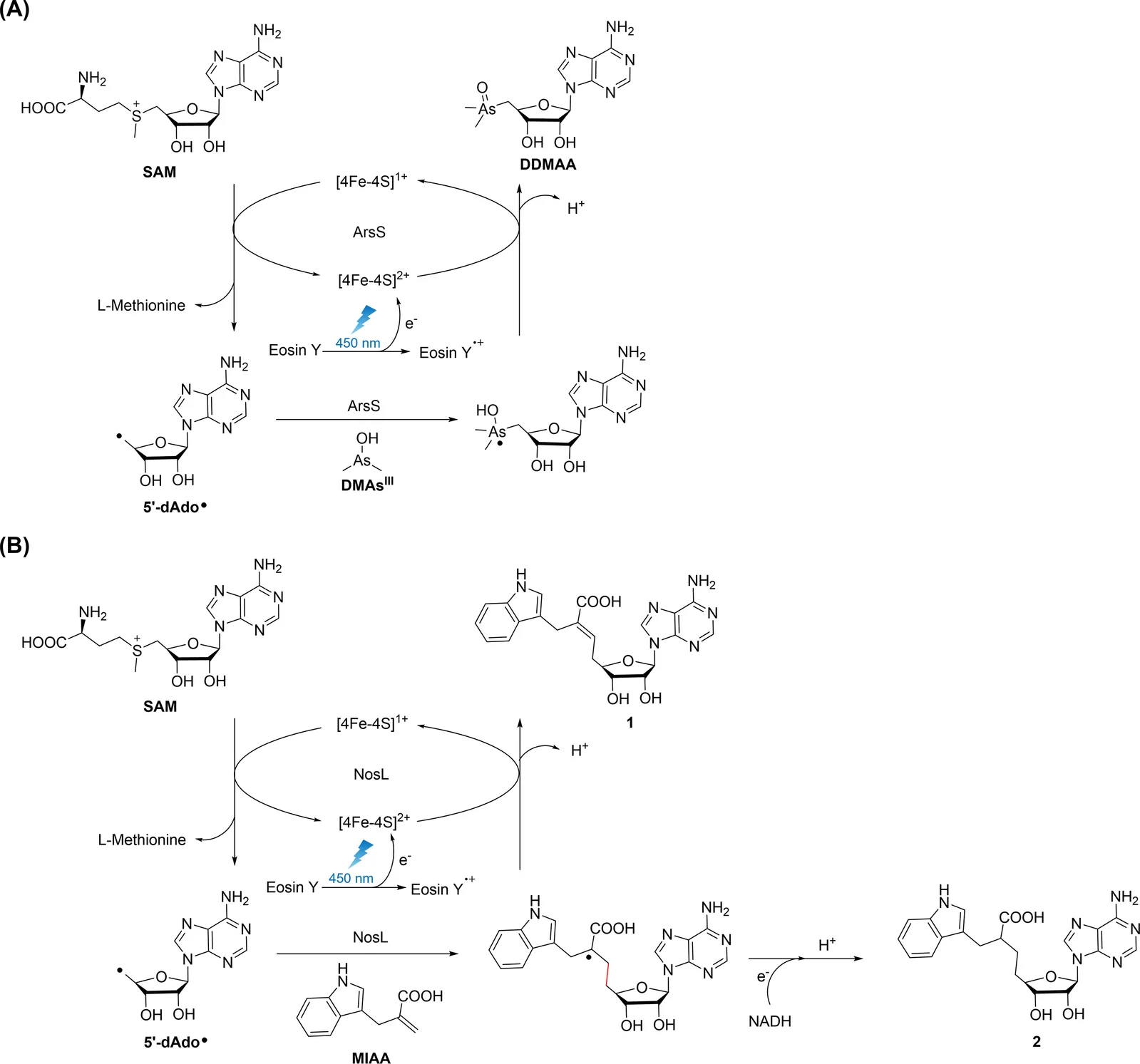
Redox-neutral rSAM-dependent reactions driven by the Eosin Y system. (A) ArsS-catalyzed adenosylation proceeds without NADH to form DDMAA. The As-centered radical intermediate reduces the [4Fe–4S]^2+^ cluster and is concomitantly oxidized to the product. (B) In the NosL-catalyzed reaction with MIAA, the radical intermediate undergoes oxidation coupled to reduction of the [4Fe–4S]^2+^ cluster, yielding the alkene product 1. In the presence of NADH, the radical intermediate instead undergoes one-electron reduction followed by protonation to form the alkane product 2.

Despite these advantages, the potential limitations of artificial electron-delivery systems for rSAM catalysis remain insufficiently explored. The use of strongly reducing photosensitizers could, in principle, promote nonproductive electron-transfer pathways involving the Fe–S cluster, auxiliary redox cofactors, substrates, or other reaction components. However, such effects have not been systematically investigated across rSAM enzymes and are likely to depend on the specific enzyme, photosensitizer, substrate, and reaction conditions. Thus, the compatibility of an artificial electron-delivery system should be evaluated on a case-by-case basis, with careful optimization of photosensitizer redox properties and reaction conditions to favor productive cluster reduction while minimizing undesired electron-transfer pathways.

## 4. Alkyl transfer using SAM analogues by cobalamin-dependent radical SAM methyltransferase

Radical *S*-adenosylmethionine methyltransferases (RSMTs) catalyze methylation reactions that extend beyond traditional S_N_2-type reactivity to include non-nucleophilic substrates such as unactivated sp^3^-carbons and aromatic heterocycles.^90–94^ These enzymes utilize two molecules of SAM in each catalytic cycle—one for the formation of the 5′-deoxyadenosyl radical (5′-dAdo˙) through reductive SAM cleavage, and another as a methyl donor for subsequent transfer. Based on sequence homology and mechanistic features, RSMTs are categorized into three classes. Class A enzymes (*e.g.*, RlmN and Cfr) use a methylated cysteine residue as a transient methyl carrier; Class B enzymes incorporate a cobalamin (vitamin B_12_) cofactor to transfer methyl groups to otherwise inert carbon centers; and Class C enzymes are HemN-like proteins that likely bind two SAM molecules but employ distinct catalytic strategies. Among these, Class B cobalamin-dependent RSMTs uniquely combine the one-electron reactivity of SAM with the group-transfer chemistry of cobalamin, enabling methylation of otherwise inert carbon centers.^92–94^

Efficient regeneration of SAM is critical for maintaining the activity of radical SAM enzymes, which often consume SAM stoichiometrically. The inherent instability of SAM further complicates long-term catalysis.^95^ To address this challenge, Gericke and co-workers (2023) developed a biomimetic SAM-regeneration platform capable of recycling SAM from its byproducts, *S*-adenosylhomocysteine (SAH) and 5′-deoxyadenosine (5′-dA), through a polyphosphate-driven adenine salvage cycle (Fig. 7A).^96^ This system was demonstrated to be compatible with class B RSMTs, including the cobalamin-dependent glutamine *C*-methyltransferase (QCMT) from *Methanoculleus thermophilus*,^97^ which requires two SAM molecules per catalytic cycle (Fig. 7B). However, the regeneration efficiency remained modest for the rSAM system, with TTNs of approximately 20, lower than those achieved with conventional methyltransferases in the same study. Moreover, only the adenine moiety of the SAM-derived byproducts is recycled, while the other components required for SAM regeneration must be supplied externally. Thus, although this biomimetic salvage strategy represents an important advance, efficient and complete SAM recycling for rSAM catalysis remains an unresolved challenge. Notably, the system also supports the biosynthesis of SAM analogues, such as *S*-adenosyl-l-ethionine (SAE), through the addition of l-ethionine (Fig. 7A). Using SAE, the QCMT-catalyzed reaction achieved ethylation of the peptide substrate (Fig. 7B), albeit with a low yield of approximately 7%. This ability to generate SAM analogues nevertheless expands the utility of the regeneration platform for accessing structurally diversified products through rSAM catalysis.

**Fig. 7:**
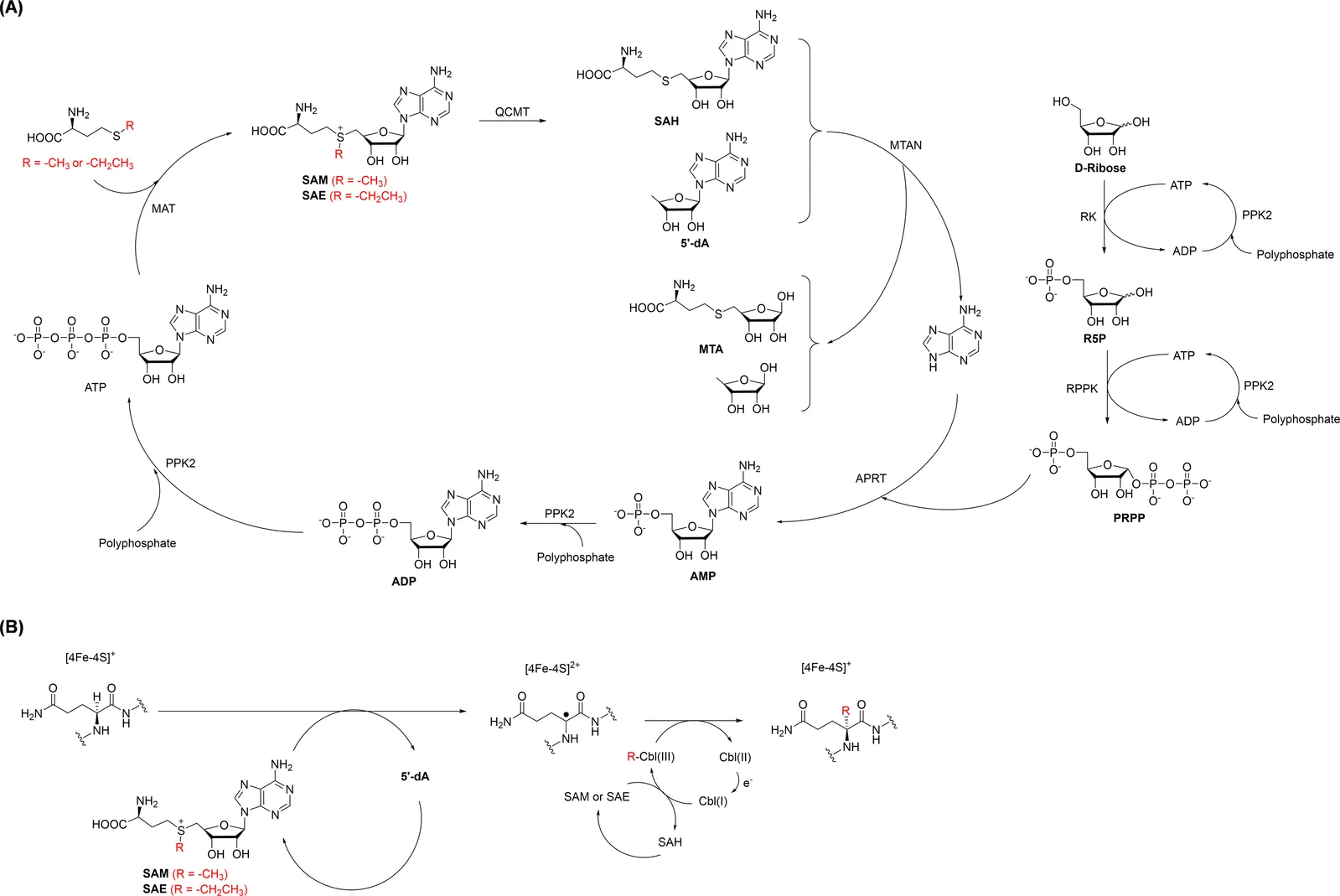
(A) Regeneration of SAM through a polyphosphate-driven adenine salvage pathway that recycles the adenine moiety of SAM-derived byproducts, including SAH and 5′-dA. Supplementation with l-ethionine instead enables the biosynthesis of SAE. (B) The regenerated SAM and SAE are utilized by QCMT for methylation and ethylation of the peptide substrate, respectively.

Building on these advances in SAM analogue engineering, two recent studies demonstrated that cobalamin-dependent RSMTs can harness a synthetic SAM analogue to catalyze fluoromethylation reactions.^98,99^ In these studies, the fluorinated SAM analogue (F-SAM) was generated *in situ* from *S*-adenosyl-l-homocysteine (SAH) and fluoromethyl iodide (CH_2_FI) using halide methyltransferase (HMT) (Fig. 8A).^100^ These enzymes employ two F-SAM molecules per catalytic cycle: one for the 5′-deoxyadenosyl radical generation and another for cobalamin fluoromethylation, forming a fluoromethylcobalamin (FMeCbl) intermediate that mediates homolytic fluoromethyl transfer to the substrate-derived radical, significantly expanding the chemical repertoire of radical SAM methyltransferases. Multiple cobalamin-dependent RSMTs, including OCMT,^97^ Mmp10,^101^ GenD1,^102^ CysS,^103^ and TokK,^104^ were shown to catalyze C–CH_2_F bond formation at unactivated sp^3^ carbon centers (Fig. 8B). The formation of the 5′-deoxyadenosine (5′-dA) byproduct and the FMeCbl intermediate was confirmed by complementary analytical methods, supporting a radical fluoromethyl transfer mechanism analogous to native methylation.

**Fig. 8:**
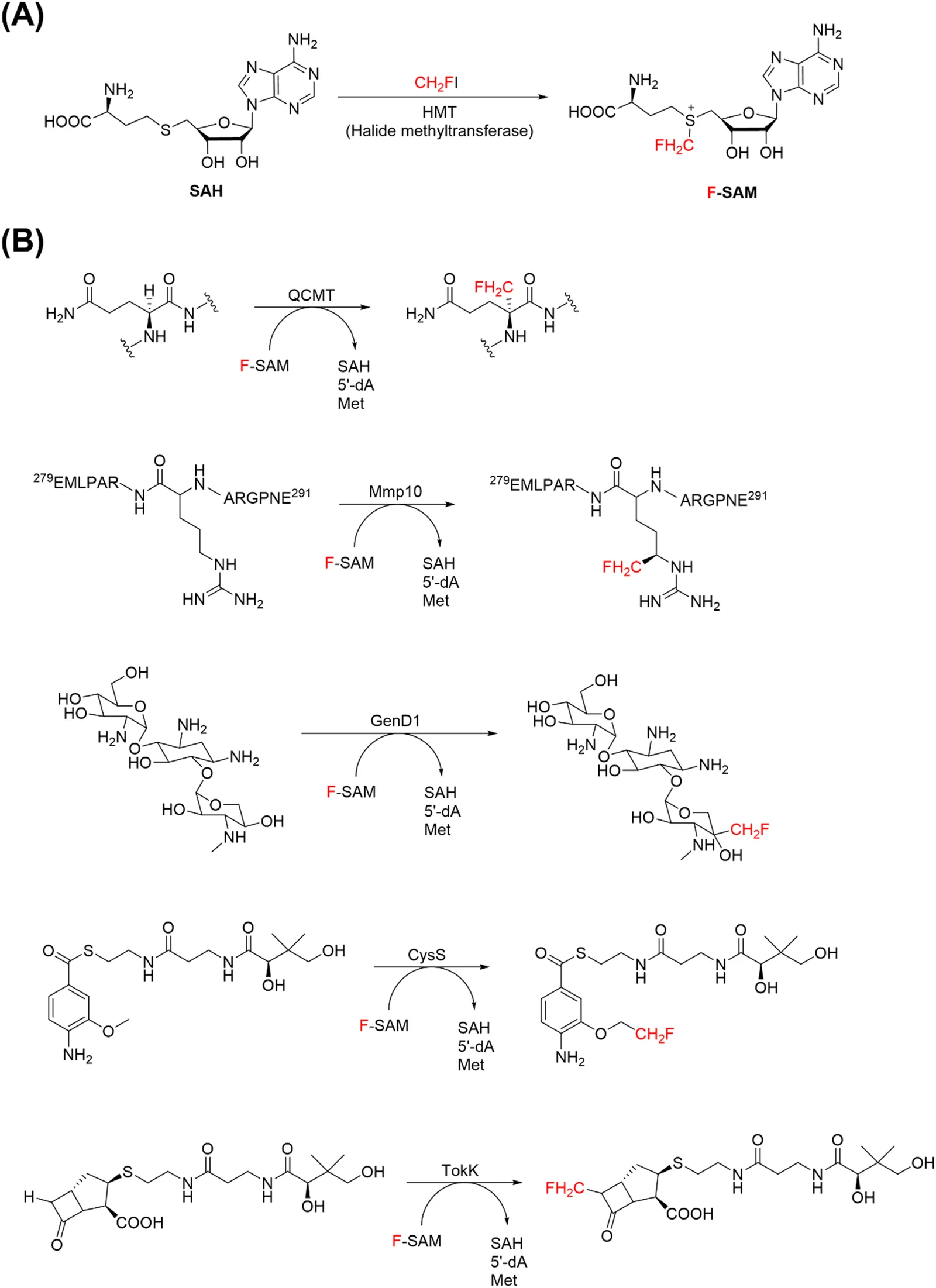
(A) Fluorinated SAM analogue (F-SAM) is synthesized from *S*-adenosyl-l-homocysteine (SAH) and fluoromethyl iodide (CH_2_FI) catalyzed by halide methyltransferase (HMT). (B) Multiple cobalamin-dependent RSMTs, including QCMT, Mmp10, GenD1, CysS, and TokK, catalyze C–CH_2_F bond formation at unactivated sp^3^ carbon centers using *in situ*–synthesized F-SAM. These results indicate that F-SAM functions both as a 5′-deoxyadenosyl radical precursor and as a viable cobalamin substrate.

While the *in situ* generation of F-SAM enables radical fluoromethylation, its inherent chemical instability resulting from depurination and carboxyl-mediated intramolecular cyclization remains a significant limitation for overall biocatalytic efficiency. Previous efforts to address this instability led to the development of stabilized fluoromethyl SAM analogues, such as FMeTeSAM^105^ and F-dcSAM^106^ (Fig. 9); however, these analogues are limited to nucleophilic methyltransferases and fail to undergo radical cleavage by cobalamin-dependent RSMTs, such as CysS, Mmp10, and QCMT. To overcome this barrier, a novel stable analogue, 7-deazaadenine-tetrazole-substituted F-SAM (F-7dz-tSAM) (Fig. 9),^107^ was recently engineered *via* bioisosteric substitution of the carboxyl and base moieties. Remarkably, F-7dz-tSAM is recognized by CysS, which cleaves the analogue to generate a 7-deaza-5′-deoxyadenosyl radical that abstracts a hydrogen atom to initiate radical *C*-fluoromethylation. When evaluated in an enzymatic cascade, F-7dz-tSAM achieved significantly higher product yields than the labile F-SAM. This work demonstrates that rationally engineered SAM analogues can overcome intrinsic cofactor instability while preserving radical reactivity.

**Fig. 9:**
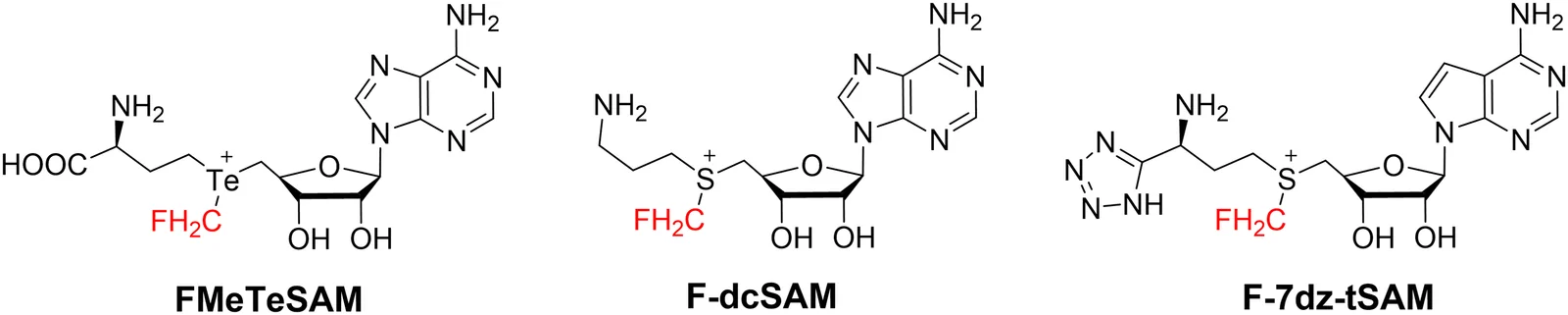
Structures of stable fluorinated SAM analogues. While FMeTeSAM and F-dcSAM fail to undergo radical cleavage by cobalamin-dependent RSMTs, the strategically engineered F-7dz-tSAM overcomes this limitation. The bioisosteric substitutions in F-7dz-tSAM prevent spontaneous degradation while retaining the necessary binding affinity to drive fluoromethylation.

Together, these studies demonstrate that engineering the SAM cofactor itself provides a powerful strategy for expanding the chemistry accessible to cobalamin-dependent RSMTs. Beyond native methylation, these enzymes can now catalyze ethylation and fluoromethylation while maintaining their radical catalytic framework, establishing SAM analogue engineering as a versatile platform for developing new-to-nature radical transformations.

## 5. New-to-nature chemistry and biotechnological applications of radical SAM enzymes

In addition to manipulating radical initiation and cofactor reactivity, exploiting the inherent substrate tolerance of rSAM enzymes provides another strategy for expanding their biotechnological applications. Recent work by Bandarian and co-workers demonstrated that the thioether-forming rSAM maturase PapB exhibits broad substrate tolerance and can catalyze peptide macrocyclization across structurally diverse substrates.^108^ In addition to native-like sequences, PapB accepts peptides containing d-amino acids, β-amino acids, and *N*-methylated residues, including substrates with extensive incorporation of noncanonical building blocks. This catalytic flexibility highlights the potential of rSAM maturases as platforms for generating structurally diverse macrocyclic peptides. Collectively, such substrate flexibility and deliberate engineering can expand the utility of rSAM enzymes for abiological transformations and biotechnological applications.

### 5.1. New-to-nature and preparative C–As bond formation catalyzed by ArsL

Arsinothricin (AST) has been characterized as a broad-spectrum antibiotic active against both Gram-positive and Gram-negative bacteria.^109^ AST targets prokaryotic glutamine synthetase with high specificity while exhibiting markedly lower affinity for the human enzyme, thereby inhibiting diverse bacterial pathogens with minimal cytotoxicity toward human cells.^109^ Moreover, AST has demonstrated promising antimalarial activity by suppressing parasite proliferation in the bloodstream and blocking transmission to mosquitoes.^110^ Since its discovery, the biosynthetic pathway and enzymatic mechanism have been extensively investigated. ArsL from *Burkholderia gladioli* GSRB05 represents a distinct noncanonical radical SAM enzyme that catalyzes C–As bond formation through SAM cleavage at the C_γ_–S bond, producing MTA and an ACP radical, which subsequently adds to As(iii) to yield the intermediate AST-OH (Fig. 10A).^85,86^ The formation of the ACP radical was confirmed by deuterium incorporation into the byproduct homoalanine when reactions were performed in D_2_O buffer.^87^ Substrate scope studies revealed that ArsL acts on trivalent As substrates, whereas pentavalent As substrates can be reduced *in situ* by dithiothreitol (DTT).^87^ This was demonstrated by the observation that pre-reduced ArsL could not convert dimethylarsenate (DMAs^V^) to AST-Me in the absence of DTT (Fig. 10B).

**Fig. 10:**
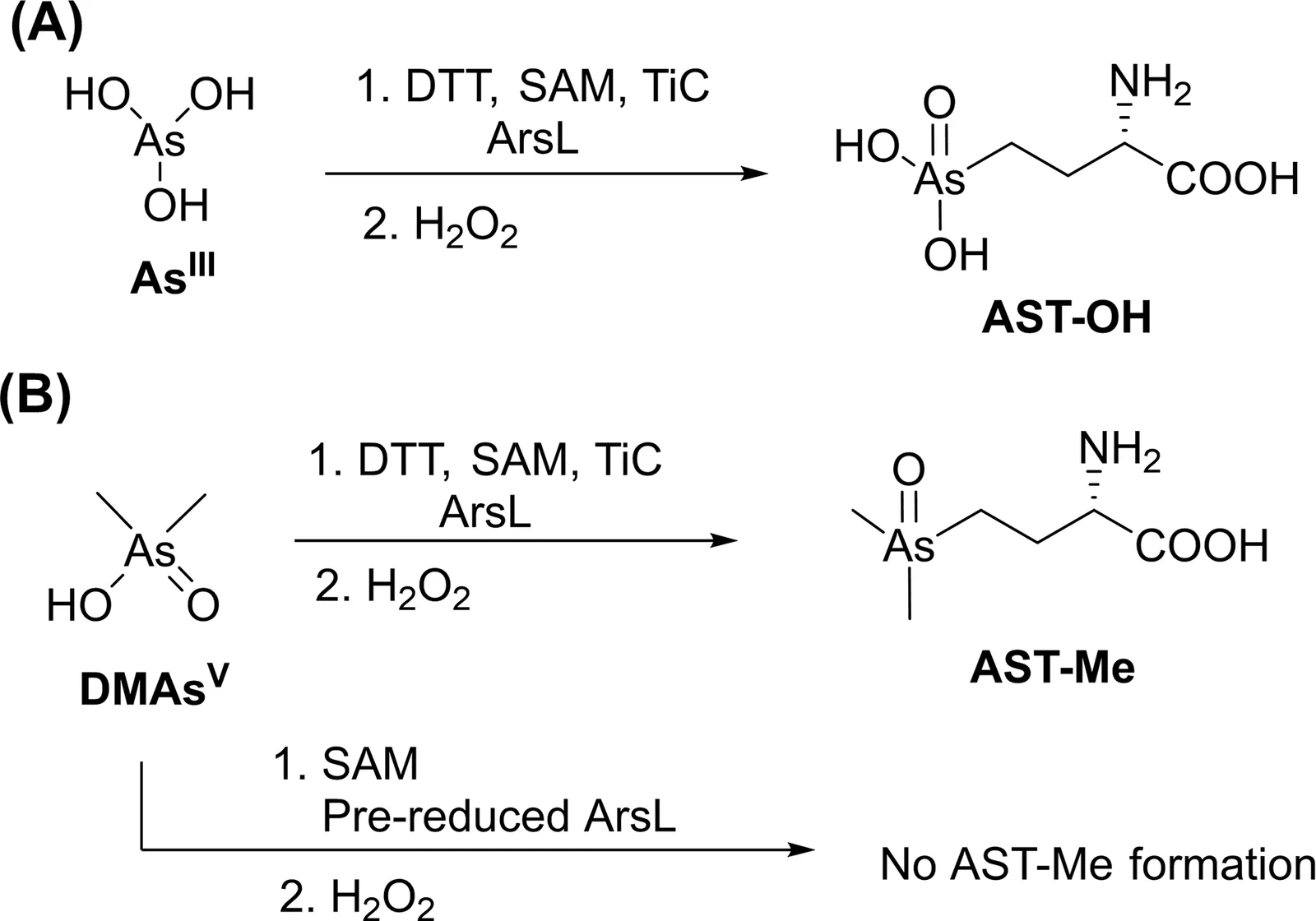
(A) ArsL-catalyzed production of AST–OH from the trivalent arsenic substrate (As^III^) and SAM. (B) ArsL does not act on pentavalent arsenic substrates, as demonstrated by the lack of activity toward DMAs^V^ in the absence of DTT when using pre-reduced ArsL, indicating that *in situ* reduction of As(v) to As(iii) is required for enzymatic turnover. TiC, titanium(iii) citrate.

The production of AST-Me from DMAs^V^ highlights a possible straightforward and efficient route for synthesizing AST from methylarsenate (MAs^V^), a bulk agricultural chemical. Additionally, the RCCLKC motif at the *C*-terminus of ArsL mediates substrate binding *via* three cysteine residues that coordinate to As(iii).^86,87^ In mutagenesis studies (Fig. 11), the M_CCA_ mutant, which likely suppresses undesired disulfide cascade formation, exhibited the highest catalytic efficiency, achieving >99% substrate conversion with over 200 total turnover numbers (TTN) using 10 mM MAs^V^,^87^ representing an approximately 100-fold increase relative to the previously reported value.^86^ Building upon this success, cell lysate containing the overexpressed M_CCA_ variant with *in vitro* [4Fe–4S] reconstitution was used for semi-preparative-scale AST synthesis from 2 mM MAs^V^ in a 250 mL reaction volume, yielding approximately 0.1 g of AST (quantitative yield) in 3 hours.

**Fig. 11:**
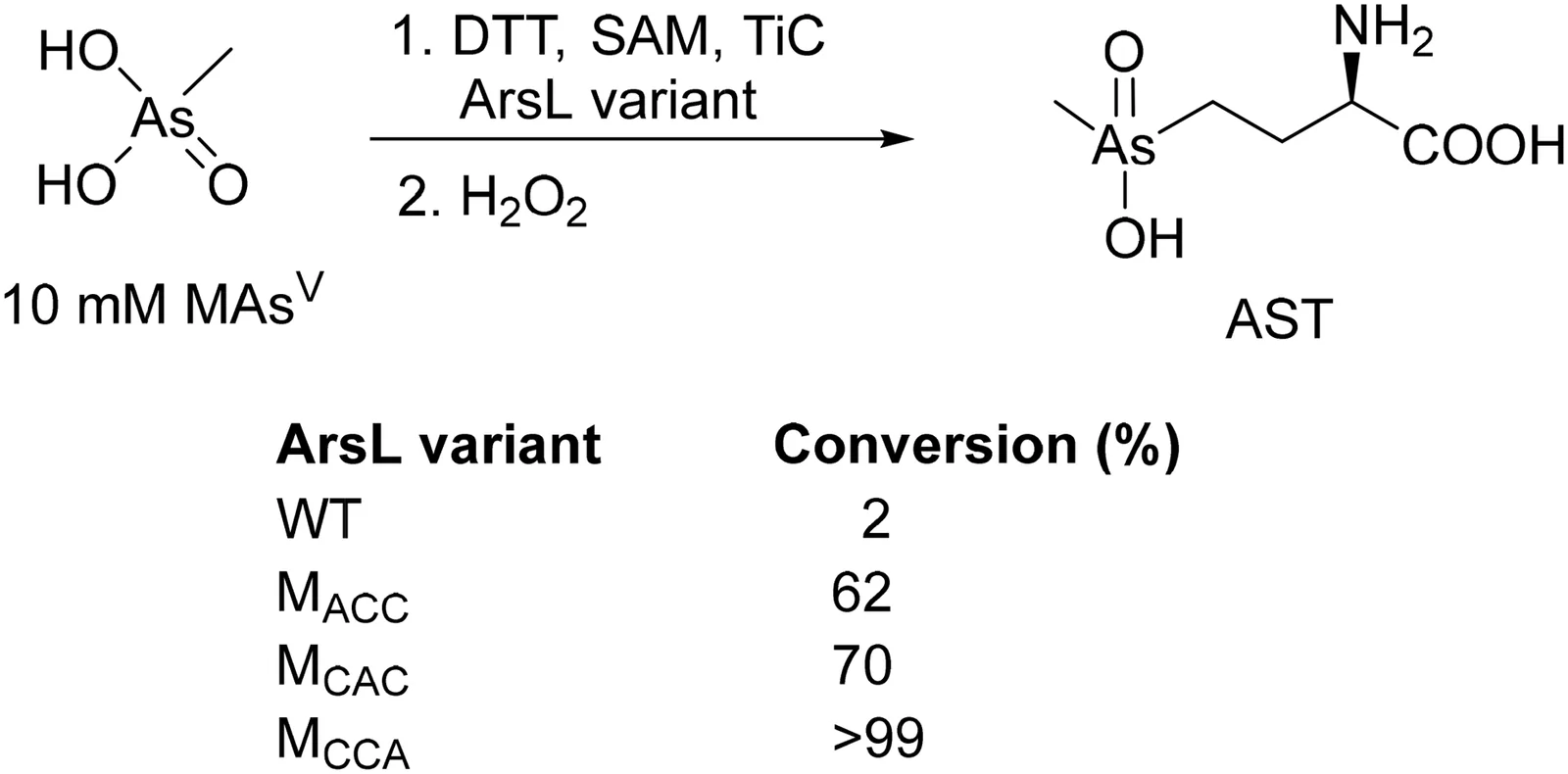
Comparison of AST production by ArsL and its mutants, showing substrate conversion using 10 mM MAs^V^.

### 5.2. Light-driven trifluoromethylation catalyzed by NosL

NosL, a tryptophan lyase from *Streptomyces actuosus*, was initially characterized for catalyzing the cleavage of l-tryptophan to produce 3-methyl-2-indolic acid during nosiheptide biosynthesis.^71–73^ A recent study revealed that NosL can be photochemically activated under blue-light irradiation in the presence of the chemical photosensitizer eosin Y, catalyzing its native reaction with significantly higher yield than that obtained using dithionite as the reductant (Fig. 5B).^63^ Previous mechanistic investigations have demonstrated the catalytic promiscuity and substrate tolerance of NosL.^88,89^ Building on this foundation, the light-driven system was subsequently applied to NosL-catalyzed trifluoromethylation of l-Trp at the C2 position using Togni reagent II or the Umemoto reagent as the trifluoromethyl donor (Fig. 12).^63,111^ However, the initial yield of 2-trifluoromethyl-Trp (Tf-Trp) was low. Screening of photocatalysts identified tris(bipyridine)ruthenium(ii), [Ru(bpy)_3_]^2+^, as an effective photosensitizer for this transformation.^63,112,113^ To probe the mechanistic role of the [4Fe–4S] cluster, the C95A mutant, which disrupts cluster coordination, was employed and completely abolished product formation. Moreover, the addition of SAM to the reaction markedly reduced the efficiency of trifluoromethylation, suggesting that SAM competes with the Umemoto reagent for binding within the active site.

**Fig. 12:**
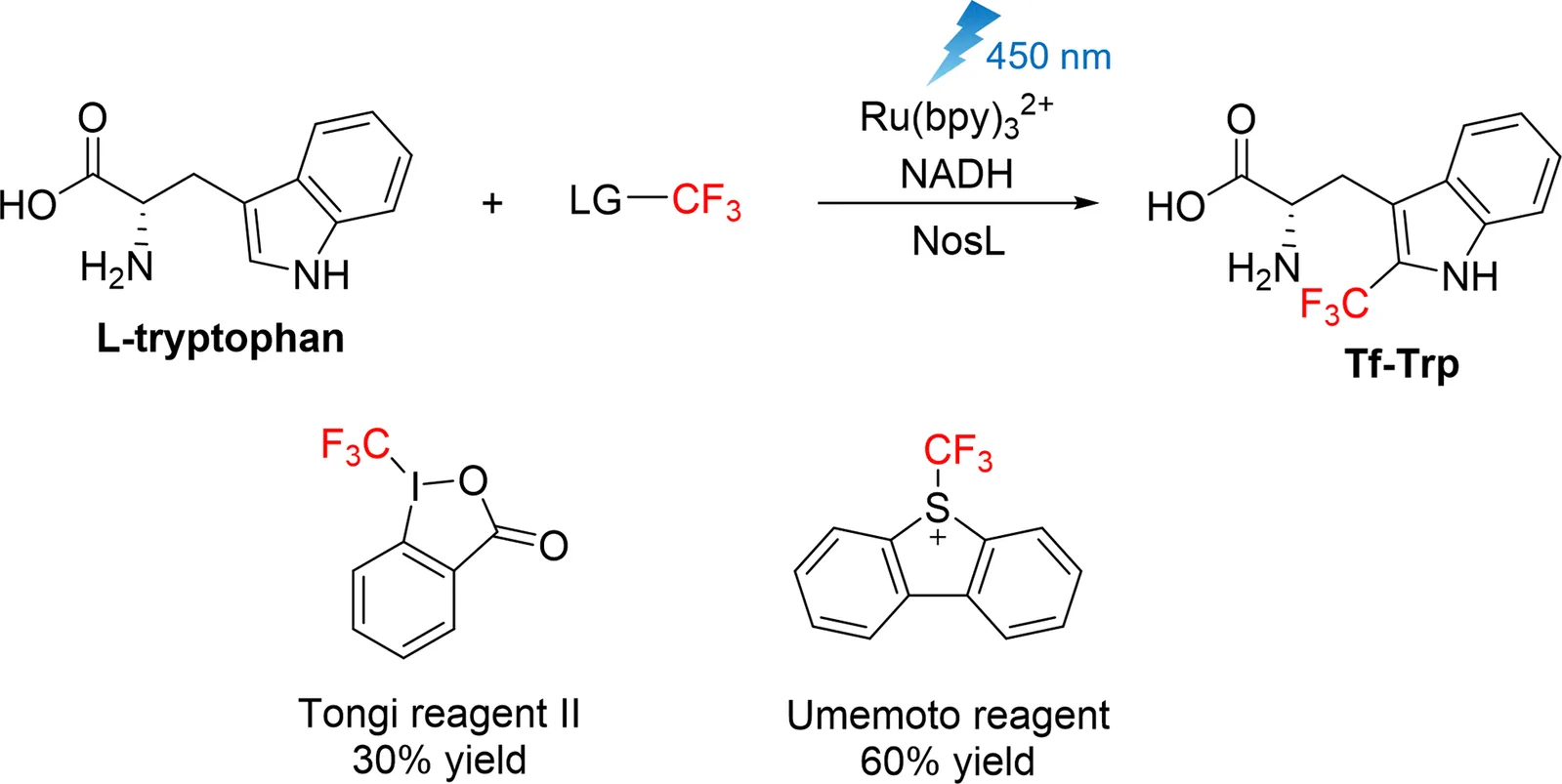
Photocatalytic trifluoromethylation of l-tryptophan catalyzed by the rSAM enzyme NosL. Under 450 nm irradiation, the photosensitizer Ru(bpy)_3_^2+^ facilitates reduction of the [4Fe–4S]^2+^ cluster, initiating the NosL-catalyzed radical reaction in the presence of NADH. This reaction enables C–CF_3_ bond formation at the C2 position of the indole ring to afford product Tf-Trp, using Togni reagent II or the Umemoto reagent as the trifluoromethyl donor.

These findings demonstrate that NosL can function as a photoactivated radical biocatalyst, effectively coupling enzymatic radical initiation with synthetic trifluoromethylation chemistry. The use of photosensitizers provides a generalizable and tunable strategy for achieving light-controlled radical SAM reactivity, thereby expanding the catalytic repertoire of this superfamily beyond its native biochemical transformations.

### 5.3. Bioorthogonal protein labeling using radical SAM enzymes

Protein labeling is an important strategy in chemical biology for the selective visualization, detection, and functional modification of proteins in complex biological systems.^114,115^ In this context, bioorthogonal methods are especially valuable because they enable selective chemical reactions on target proteins without perturbing native cellular processes.^116,117^ Among the available reactive handles, aldehydes are particularly attractive because of their small size and efficient chemoselective reactivity in bioconjugation. Although aldehydes can be introduced by unnatural amino acid (UAA) incorporation, this approach often requires engineered translational machinery and may be limited by reduced incorporation efficiency and the synthetic accessibility of the UAA.^118,119^ A more practical alternative is the enzymatic generation of formylglycine (FGly), an aldehyde-containing amino acid that can be selectively installed into proteins and subsequently derivatized with hydrazine- or aminooxy-containing probes under mild conditions.^120,121^ Conventional aldehyde-tag technology, derived from sulfatase biosynthesis, relies on conversion of a Cys/Ser residue within the motif (*C*/*S*) × (*P*/*A*) × *R* to FGly.^122–124^ In this context, alternative enzymatic systems that act on distinct peptide sequences are of particular interest, as they broaden the scope of aldehyde-tag installation and create new opportunities for orthogonal protein labeling.

Recent work has shown that rSAM enzymes provide a promising solution for expanding conventional aldehyde-tag technology. Representative rSAM-mediated routes to FGly formation, including the AtsB- and SjiB-based aldehyde-tagging systems, are summarized in Fig. 13. One such example is the anaerobic sulfatase-maturating enzyme AtsB, an rSAM protein that converts cysteine or serine residues within aldehyde-tag motifs to FGly.^125^ In particular, AtsB from *Methanosarcina mazei* was shown to modify non-classical serine-type tags, with the SAPAR motif supporting efficient FGly formation and subsequent fluorescent labeling.^126^ However, these studies were carried out under anaerobic *in vitro* conditions. By contrast, the RiPP cyclophane-forming enzyme SjiB displays unusual catalytic promiscuity, oxidizing serine to FGly on a minimal five-residue substrate without the need for a leader peptide, and was subsequently repurposed as a dedicated FGly-generating enzyme for protein labeling.^127^ This led to the development of the YQSSI aldehyde tag, which is biosynthetically orthogonal to the classical sulfatase-derived motif and enabled protein labeling not only *in vitro* but also directly in *E. coli* cells.^128^ Together, these examples show that rSAM enzymes can expand the aldehyde-tagging toolbox for bioorthogonal protein labeling.

**Fig. 13:**
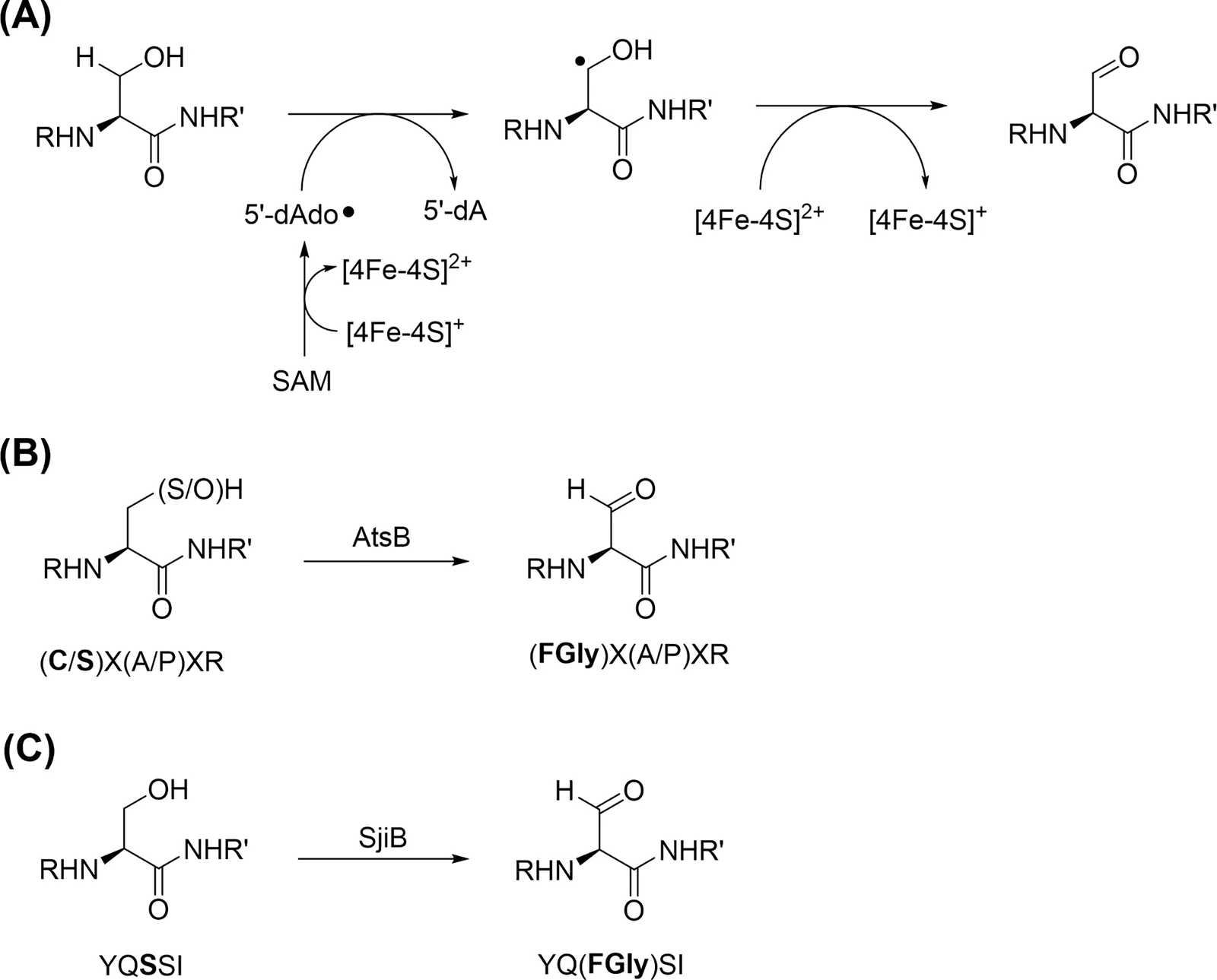
Radical SAM enzyme-mediated formation of formylglycine (FGly) for aldehyde-tag-based protein labeling. (A) General mechanism of FGly formation by radical *S*-adenosylmethionine (rSAM) enzymes, illustrated for serine oxidation. Reductive cleavage of SAM by the [4Fe–4S] cluster generates the 5′-deoxyadenosyl radical, which initiates hydrogen abstraction from the serine side chain, followed by oxidation to yield the aldehyde-containing FGly residue with concomitant reduction of another [4Fe–4S] cluster. (B) AtsB-catalyzed conversion of cysteine- or serine-containing aldehyde-tag motifs to FGly. (C) SjiB-catalyzed conversion of the serine residue in the minimal peptide tag YQSSI to the orthogonal FGly-containing aldehyde tag YQ(FGly)SI.

## 6. Outlook

In Nature, rSAM enzymes catalyze a remarkably diverse range of chemical transformations. However, their development as practical platforms for biocatalysis and chemical biology remains at an early stage. Recent advances in photochemical activation, SAM analogue design, and the discovery of noncanonical radical reactions have begun to expand their catalytic repertoire beyond native biological functions. Nevertheless, substantial challenges remain before these enzymes can be routinely used in practical biocatalytic applications.

A major practical limitation of rSAM catalysis is its dependence on oxygen-sensitive Fe–S clusters, whose proper assembly and maintenance are essential for activity. Efficient Fe–S cluster incorporation during heterologous expression often requires co-expression of Fe–S biogenesis systems, such as ISC or SUF, from an additional plasmid; alternatively, the clusters can be reconstituted *in vitro* under anaerobic conditions using purified apo-proteins. Purification and handling of rSAM enzymes nevertheless require carefully controlled anaerobic conditions, complicating routine manipulation and scale-up. Future efforts should therefore focus on establishing robust biocatalytic systems that maintain catalytic activity under practically manageable anaerobic conditions. In addition, cell-lysate and whole-cell systems may provide useful alternatives by circumventing protein purification and, particularly in whole cells, enabling continued support from engineered Fe–S biogenesis and cellular redox systems.

Efficient electron delivery and SAM supply also remain important challenges. Electron-delivery systems must support productive reduction of the [4Fe–4S] cluster to sustain catalysis, while sufficient SAM availability is required for continued catalytic turnover. Recent engineering of an *E. coli* strain with a short-circuited SAM cycle, in which the intracellular SAM pool is maintained through direct methylation of SAH using a synthetic methyl donor, illustrates the potential of metabolic engineering for SAM-dependent whole-cell biocatalysis.^129^ Although this strategy has not yet been demonstrated for rSAM enzymes, it offers a potential new avenue for whole-cell rSAM catalysis. Improved SAM-regeneration strategies will also be important for biocatalysis using purified rSAM enzymes or cell lysates, particularly when SAM analogues are required. Protein engineering offers promising routes to improve catalytic efficiency, selectivity, and substrate scope, thereby broadening the utility of rSAM enzymes for non-native transformations. Progress in these areas will be important for moving rSAM biocatalysis beyond proof-of-concept studies and toward increasingly practical synthetic applications.

## Author contributions

The manuscript was written through contributions of all authors. All authors have given approval to the final version of the manuscript.

## Conflicts of interest

There are no conflicts to declare.

## Data Availability

No primary research results, software or code have been included, and no new data were generated or analysed as part of this review.
